# Open-source low-cost design of a buoy for remote water quality monitoring in fish farming

**DOI:** 10.1371/journal.pone.0270202

**Published:** 2022-06-22

**Authors:** Juan D. Medina, Alejandro Arias, Juan M. Triana, Luis F. Giraldo, Fredy Segura-Quijano, Andres Gonzalez-Mancera, Andres F. Zambrano, Julián Quimbayo, Eduardo Castillo

**Affiliations:** 1 Department of Electrical and Electronic Engineering, Universidad de los Andes, Bogotá, Colombia; 2 Department of Mechanical Engineering, Universidad de los Andes, Bogotá, Colombia; 3 Department of Systems Engineering, Corporación Universitaria del Huila, Neiva, Colombia; 4 Department of Veterinary Medicine, Corporación Universitaria del Huila, Neiva, Colombia; University of Glasgow, UNITED KINGDOM

## Abstract

In this paper we present the design of an open-source and low-cost buoy prototype for remote monitoring of water quality variables in fish farming. The designed battery-powered system periodically measures temperature, pH and dissolved oxygen, transmitting the information locally through a low-power wide-area network protocol to a gateway connected to a cloud service for data storage and visualization. We provide a novel buoy design that can be easily constructed with off-the-shelf materials, delivering a stable anchored float for the IoT device and the probes immersed in the water pond. The prototype was tested at an operating fish farm, showing promising results for a low-cost remote monitoring tool that enables automatic data acquisition and storage in fish farming scenarios. All the elements of this design, including hardware and software designs, are freely available under permissive licenses as an open-source project.

## Introduction

Monitoring water-quality variables such as dissolved oxygen, temperature, and pH is key in fish farming [1, 2]. Typically, fish farmers manually collect measurements from these variables at frequencies that vary depending on the dynamics of the pond and environment [1, 3]. However, manual measurement of water-quality variables can be time-consuming and implies very low acquisition rates and frequent calibration. These limitations have motivated the development of automatic remote monitoring devices capable of reducing the burden on the fish farmer for data collection and increasing the acquisition frequency. For example, a device developed in Kenya transmits data collected from pH and temperature sensors that is subsequently processed and accessed through an app [4]. Also, in the work in [5], a wireless sensor network was developed and deployed to monitor and control an aquaculture system. Libelium, a wireless sensor network platform provider, offers several systems designed for remote monitoring of water-quality variables [6].

**Fig 1:**
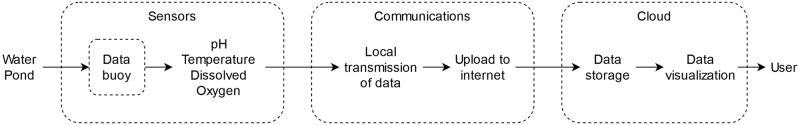
Diagram of the designed water quality monitoring device.

Even though these solutions are proven to work in a wide variety of scenarios in fish farming, they are either expensive for small-scale fish farmers and would not be affordable in lower-income countries or not open for shared development. For example, a solution provided by Libelium can be as expensive as 17 times the minimum wage in Colombia, a country in South America. There is a need to develop low-cost devices for remote monitoring of water quality variables in fish farming in a joint effort of the research community. In this paper, we present a system for remote water quality monitoring in fish farming scenarios that uses low-cost equipment, has low-power consumption, is scalable, and is open-source to continue being developed by the research community in the field. The developed prototype is a battery-operated data buoy for measuring temperature, dissolved oxygen and pH with the possibility to extend to other water quality variables, as shown in Fig 1. These measurements are transmitted using LoRaWAN with services from The Things Network (TTN) [7] to an on-site gateway connected to the Amazon Web Services (AWS) [8] cloud for storage and visualization by the user. This prototype involves a novel buoy design constructed using off-the-shelf materials and provides stable flotation that supports the electronics and the probes immersed in the water pond. The entire design has been released as an open-source project and uses commercially available products.

The rest of the document is organized as follows. First, each device component is described, including the electronics and mechanical structure of the buoy. Then, the completed prototype and field test results are presented. This is followed by a discussion on the various future potential research directions to improve this design. Finally, the last section concludes the work.

## Materials and methods

In the following sections we present the detailed design of the monitoring system as presented in the diagram in Fig 1. A complete guide to present the documentation, software and hardware designs including circuit schematics and PCB fabrication files can be found in GitHub via https://github.com/open-pisciculture/open-source-fish-farming-prototypes.

### Buoy design

**Fig 2:**
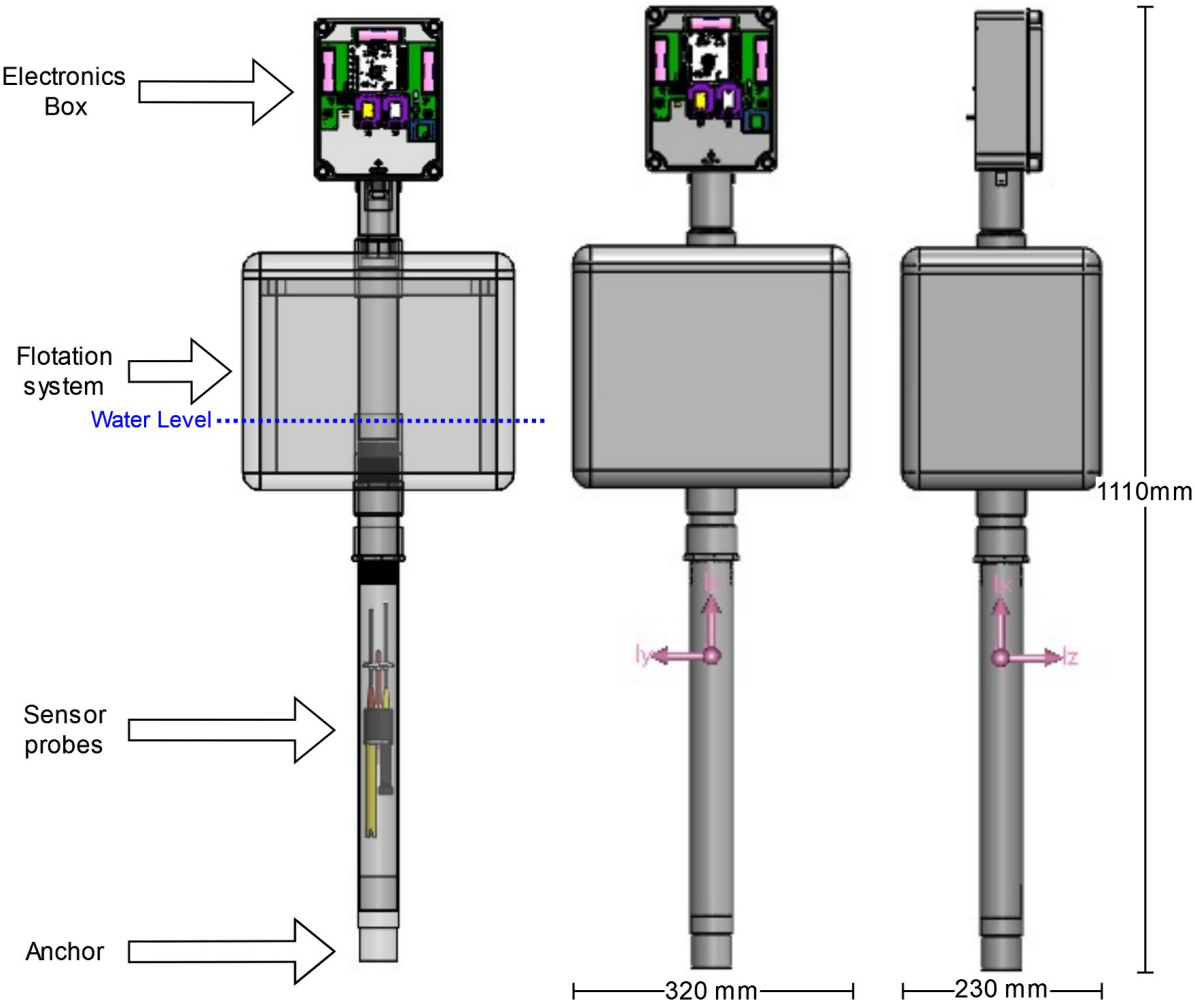
Buoy prototype diagram indicating each section.

The data buoy includes the electronics for data transmission, the sensor probes for measurement, and the mechanical structure for flotation and protection from the environment. The system must shield the electronics from conditions such as rain and dust. In addition, the sensor probes must be immersed at a specific depth in the water pond while still allowing for adequate water flow. The buoy has to be stable with respect to wind and water movement. Fig 2 shows each of the data buoy sections, alongside the dimensions and center of gravity location.

The following design was developed according to the specified requirements. At the top of the buoy the electronics were housed inside a modified IP55 electrical box with two additional apertures. The sensor probe cables output the first aperture using a PG13.5 cable gland towards the water. The second aperture was used for antenna placement. A 10 L expanded polystyrene box was used as the flotation device and to support the electrical box. Weight was added inside the polystyrene box to locate the center of gravity below the center of buoyancy and stabilize the object when subjected to external disturbances. The sensor probes were placed at a depth of 50 cm through a tube that allows sufficient water flow, but protects them from fish and other unwanted object collisions. Finally, an anchor was added to keep the system in a fixed position. The anchor was designed to reach the bottom of the pond.

### Sensors

The device should ideally operate for long periods without human intervention, be affordable for small-scale farmers, and be easy to build without requiring specialized parts or equipment. Different sensors are available in the market: some products are cheaper but require calibration before every measurement; others incorporate more recent technology, such as optical sensors with extended periods between calibration and maintenance, but at a much higher price. We chose sensors from Atlas Scientific [9] of the evaluated commercial products, which proved to offer the best costs and quality trade-off. These sensors require occasional calibration and maintenance only every few months, and they are affordable. Table 1 summarizes the characteristics of each sensor.

**Table 1:** Characteristics of the used water quality sensors. Variable *t* refers to temperature.

Variable	Sensor	Range	Accuracy	Time before recalibration	Life expectancy	Price
Temperature	PT-1000 Temperature Probe	-55 to 125°C	±(0.15 + (0.002 ⋅ t))	-	15 Years	∼ $ 23.99
pH	Lab Grade pH Probe	0–14	± 0.002	∼ 1 Year	∼ 2.5 Years +	∼ $ 85.99
Dissolved Oxygen	Lab Grade D.O. Probe	0–100 mg/L	± 0.05 mg/L	∼ 1 Year	∼ 4 Years	∼ $ 243.99

### Communications

The device must regularly transmit data from sensors, have enough range to cover vast land areas, be low-cost and reach possibly years of battery life. Communication technologies range from the more traditional Wi-Fi and Bluetooth to more recent ones such as low-power wide-area networks (LPWAN) and innovative satellite solutions. LPWANs are designed for low-power long-range communications with low bit rates, such as transmitting sensor values from battery-operated devices [10]. Satellite constellations, such as Swarm Technologies [11] and Lacuna Space [12] promise to eliminate the need for on-site internet connection by offering a network based on satellites. These are much newer technologies still in development and, in most cases, are available only in select countries and through beta programs. Due to this, they were not considered in this work.

For this prototype, LoRaWAN was chosen due to its low power consumption, high range and low cost. It can cover ranges up to 20 km and reach long lifetimes with batteries [10]. The main limitation of this technology is its low bit-rate, which is around the kilobit or even bit per second range. In this case, since sensor values are measured between long time intervals and correspond to a payload of a few bytes, LoRaWAN is an appropriate fit for the application. In the Field test section we discuss the selection of the sampling time for this environment.

### Hardware

**Fig 3:**
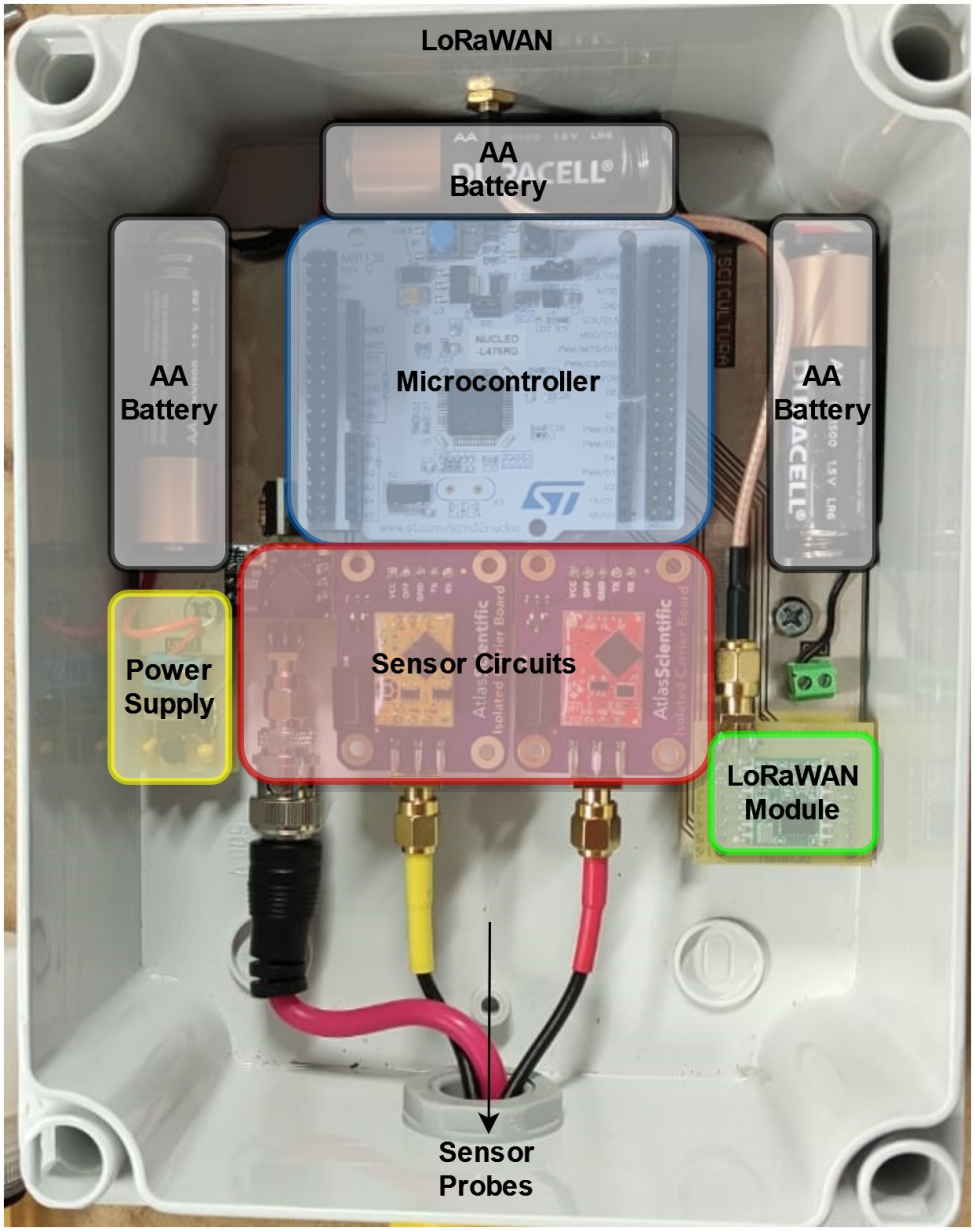
Developed circuit board.

The device electronics must run on batteries for an extended amount of time. Accordingly, the circuit must use low-power components and circuitry to read the sensor probes and transmit the data using LoRaWAN. The developed circuit board is illustrated in Fig 3, where each of the main sections are shown in colors. Following, we describe each one of the hardware components.

#### Power supply

Three AA batteries in series feed into an MCP1700 low dropout voltage regulator for the 3.3 V power supply. This chip was chosen due to its low quiescent current of 2 *μ*A and 250 mA maximum current. In addition, the microcontroller controls the sensor’s power delivery using a MOSFET switch.

#### Microcontroller

**Fig 4:**
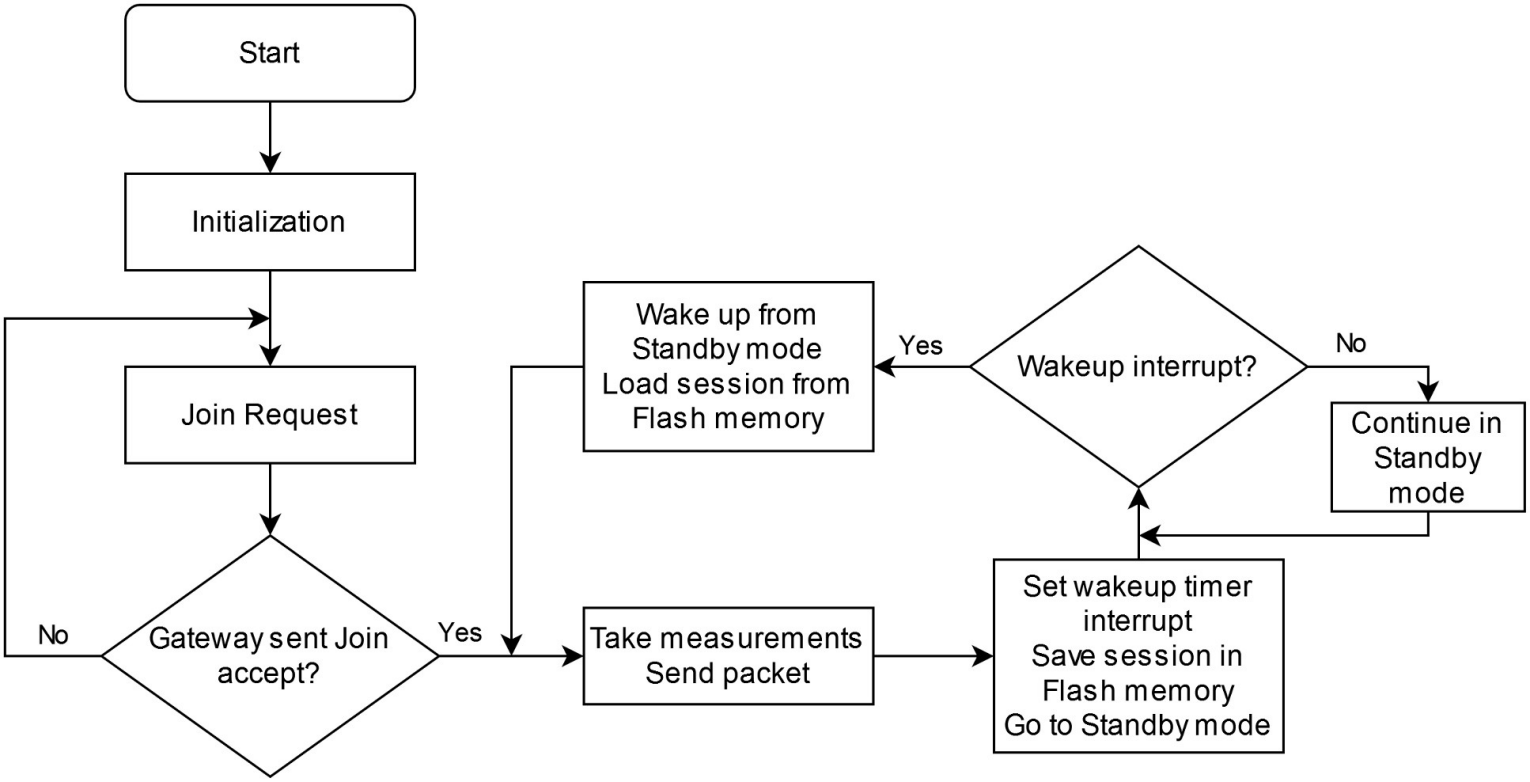
Data buoy software flowchart. Each of the steps the program takes is shown here.

The prototype uses a NUCLEO-L476RG board with an STM32L476RG microcontroller. It includes various low-power modes and incorporates both I2C and SPI peripherals to communicate with the sensor circuits and LoRaWAN module, respectively. The developed code handles the LoRaWAN communications, reads the sensor values, and reduces the power consumption. First, the chosen LoRaWAN module does not include the LoRaWAN stack. Due to this, the microcontroller must implement it to transmit data. Various libraries exist for this purpose, most notably Semtech’s LoRaMac-node [13] and arduino-lmic [14] from MCCI, which are available under open-source licenses. This project uses arduino-lmic due to its straightforward implementation in STM32 microcontrollers. Second, the microcontroller reads each sensor using the I2C protocol. Each sensor circuit was assigned a known unique I2C address beforehand to differentiate between them. Lastly, to maximize battery life, the microcontroller places itself into a low-power mode and turns off the sensor circuits during the wait periods between measurements. The microcontroller wakes up after the sampling period has passed by programming the internal RTC alarm to generate an interrupt. Fig 4 shows the flowchart of the developed code.

#### Sensor circuits

The microcontroller communicates with a reading circuit connected to each sensor probe. These circuits enable easy converting of the voltages or currents from the sensor probes into the measured value. Furthermore, the pH and dissolved oxygen reading circuits are electrically isolated with an additional isolation board. This isolation is required because the two sensors cause interference, leading the microcontroller to receive incorrect values. It is important to note that the temperature sensor does not require electrical isolation. For this reason, it does not use this additional board.

#### LoRaWAN module

Each country has different regulations for LoRaWAN that affect which specific device can be used and how it should be configured. Primarily, the frequency in which LoRaWAN can operate should be verified before choosing a module. In this case, the prototype uses an RFM95W transceiver module that transmits and receives at 915MHz. Other countries may have different regulations and thus would require a different version of the module.

### Cloud

**Fig 5:**
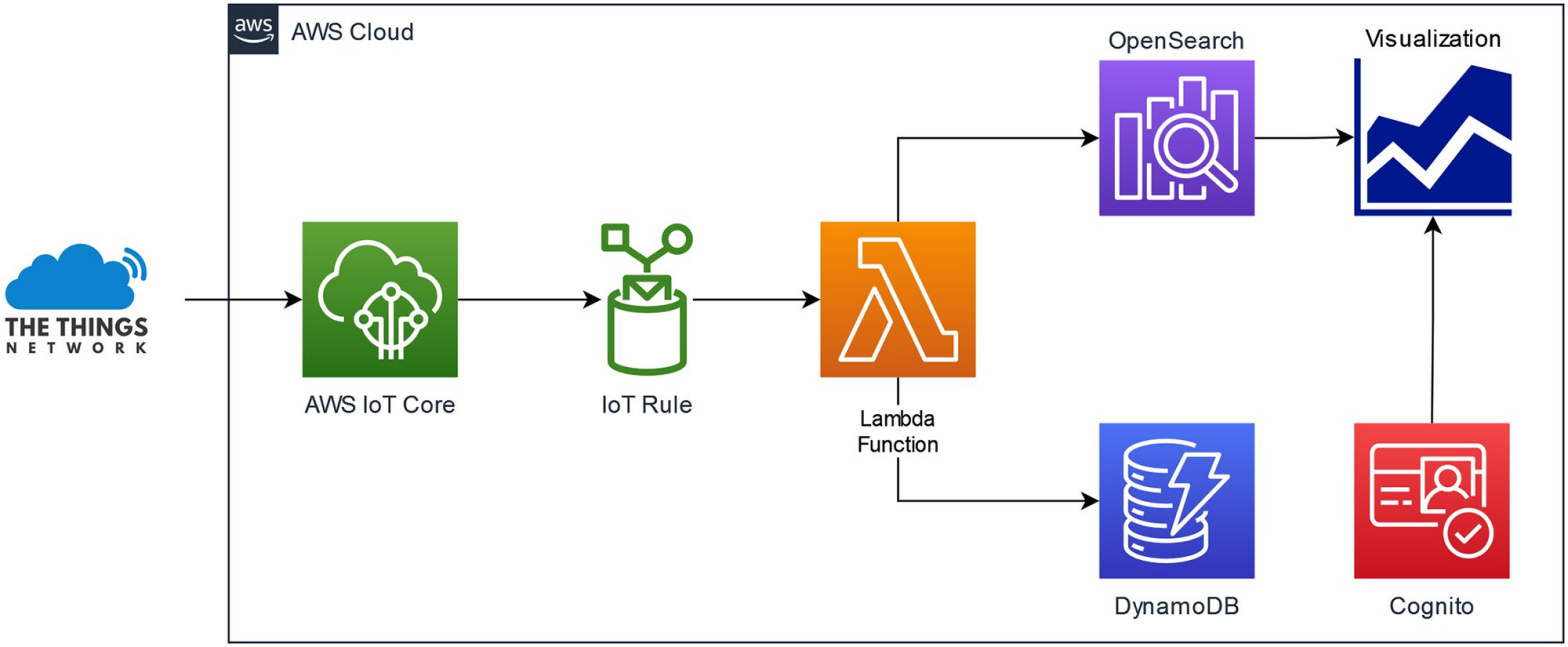
AWS services diagram.

Amazon Web Services was chosen as the cloud provider. Its main role is to receive the data from The Things Network and forward it for storage and visualization. Fig 5 shows the cloud services used in this implementation and a brief description is given below. Each service has an equivalent version in each Cloud provider. Thus, it is possible to replicate this architecture in other frameworks such as Azure or Google Cloud. The main reason for selecting AWS was because The Things Network provides an integration available as an AWS CloudFormation template.

#### AWS IoT core

It connects The Things Network platform with AWS for sending and receiving messages to and from the IoT devices.

#### OpenSearch

Provides access, handling, and storage of data. It includes, among other tools, OpenSearch Dashboards, a user interface that enables users to explore their data as time series.

#### DynamoDB

A serverless NoSQL database. Its associated costs are relatively low, fulfilling the function of storing the data received.

#### Lambda

Uploads data to databases DynamoDB and OpenSearch.

#### IoT rules

These rules give IoT devices the ability to interact with AWS services. In particular, the main functionality is triggering the Lambda function to handle the received data.

#### Cognito

It allows the system to add user sign-up, sign-in, and access control to web or mobile apps. In this case, it was implemented to manage credentials and access to OpenSearch Dashboards.

## Results

### Implemented prototype

**Fig 6:**
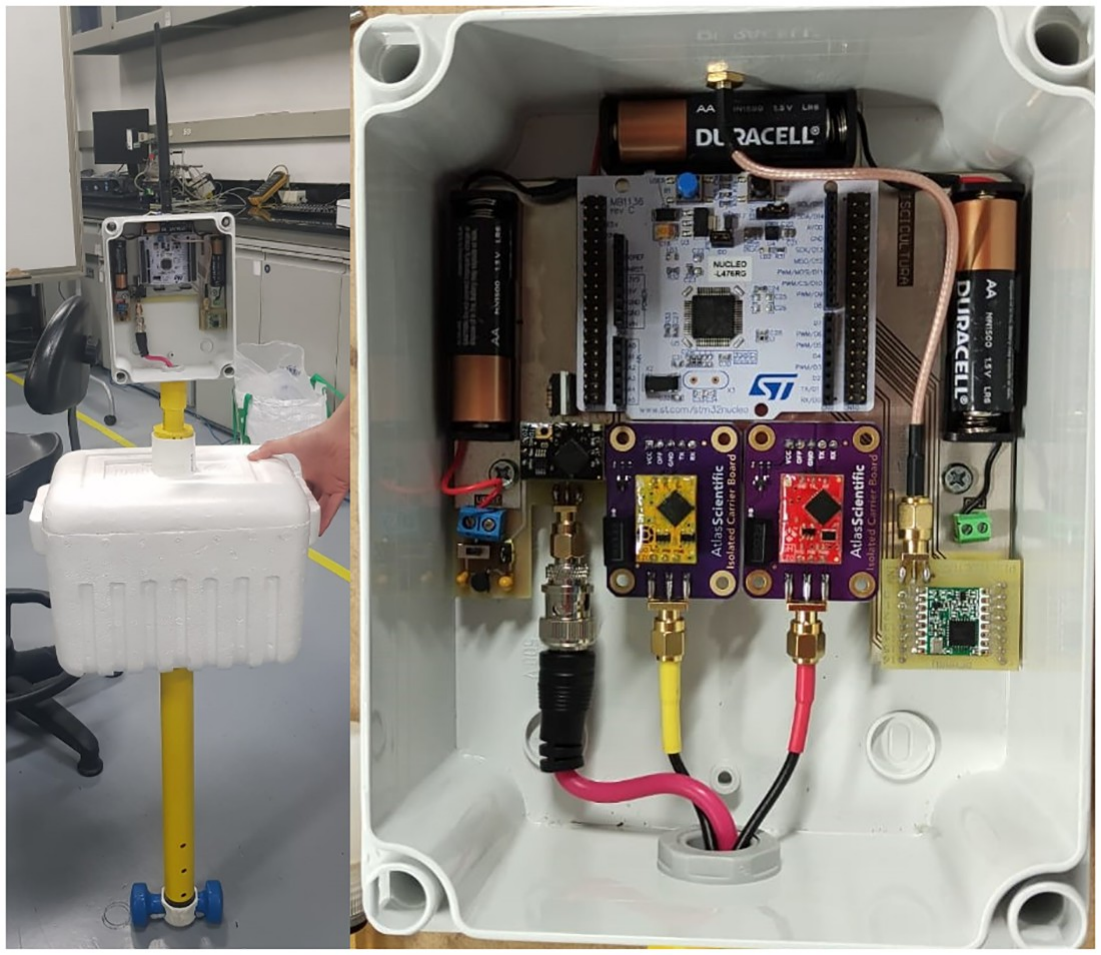
Assembled data buoy prototype.

The device was first validated under controlled conditions with a small water tank before deployment at the fish farm. The total cost of the device is approximately 658 USD. Fig 6 shows the assembled final version of the prototype.

**Fig 7:**
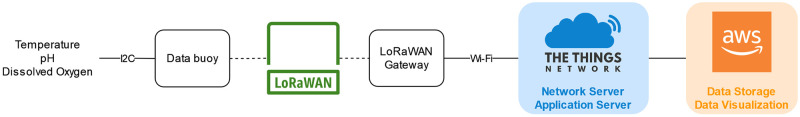
Data buoy system architecture. This diagram shows the main components of the developed system required to bring information from the pond to the user.

Fig 7 shows the entire system architecture. Data collection begins by reading the sensors and obtaining the temperature, pH and dissolved oxygen values. The data are then transmitted using LoRaWAN to the gateway located at the fish farm. If the gateway has an internet connection, the data are forwarded to The Things Network and finally sent to be stored in the AWS cloud, ready to be consulted by the user.

**Fig 8:**
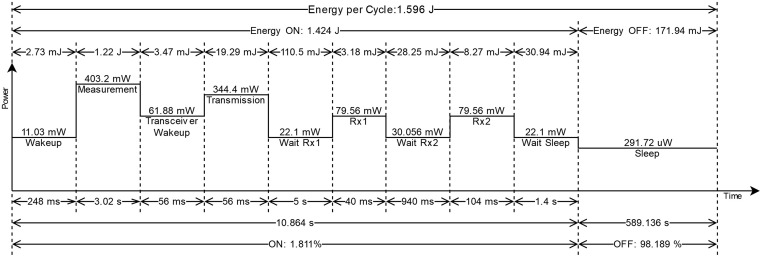
Device power consumption profile. The power consumption for a single operation cycle is shown here. The total duration of the cycle is 10 minutes. Image not to scale.

Additionally, measurements of power consumption were done for each of the states to quantify the device autonomy following the energy consumption model presented in [15]. These are summarized in Fig 8 and the data and procedure details can be found in the project repository. The total energy required by a single operation cycle of 10 minutes can thus be estimated as 1.596 J.

### Field test

The device was tested at a fish farm close to Neiva, Colombia, shown in S1 Video.

**Fig 9:**
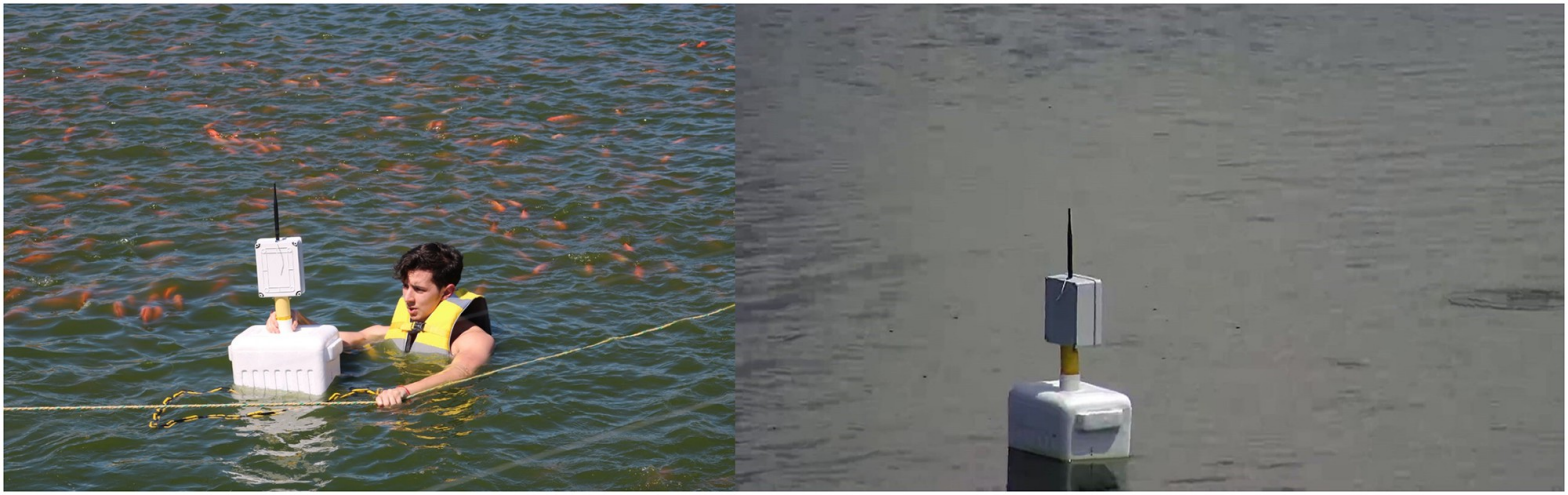
Buoy prototype operating inside the pond.

First, LoRaWAN coverage data was obtained. After that, the buoy device was deployed into the pond, and the gateway was installed in a storage room near the pond. The monitoring system was tested during seven days and remotely supervised with live camera feeds and by watching the data collected in the cloud. Fig 9 shows the device operating at the selected water pond. A video of the data buoy operation can also be seen in S2 Video. Following, we describe the results obtained during the field test.

#### Coverage map

**Fig 10:**
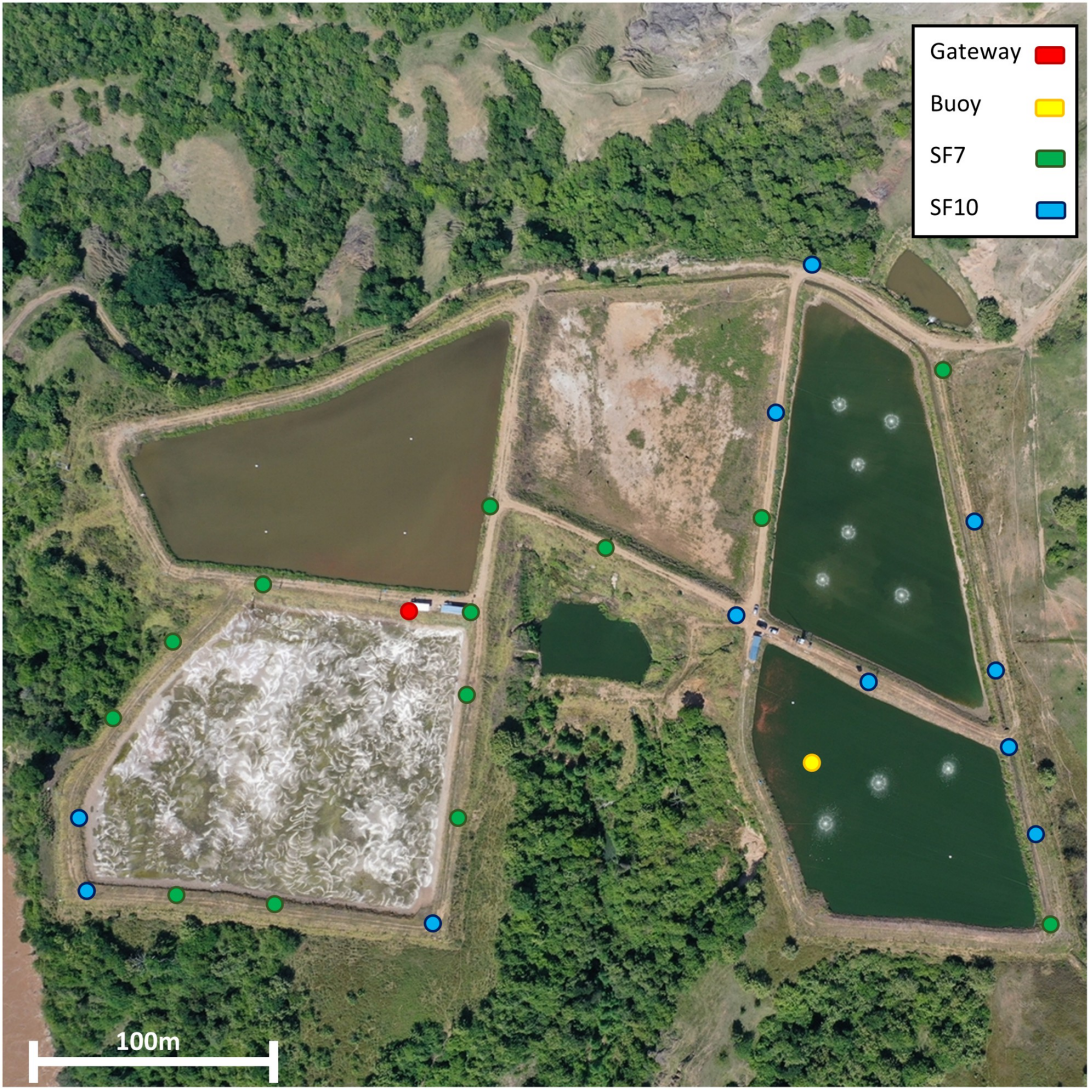
LoRaWAN coverage data on a drone image of the fish farm.

An open-source GPS LoRaWAN node prototype was developed to transmit the current coordinates. The data sent by this node are stored in the cloud along with the packet information, such as the Received Signal Strength Indicator (RSSI) and the Spreading Factor (SF). Fig 10 shows the collected data points, showing that the communication system has a good performance for this particular environment. The SF characteristic shown is of particular importance and affects the range and power consumption of the node. It represents a trade-off between range, airtime and data rate, and six different choices are available from SF7 to SF12. Particularly for this application, the energy required increases as airtime increases as well, because the device will be running for a larger amount of time. The lower values of SF, such as SF7, present a decreased power consumption, but have a shorter operation range. In contrast, higher values such as SF10, have an increased power consumption but a longer operation range. Thus, the SF is an important parameter that must be adequately chosen depending on each node’s particular environmental conditions and location [10]. In this case, SF7 is enough for transmitting the data with the chosen buoy location.

#### Internal temperature

**Fig 11:**
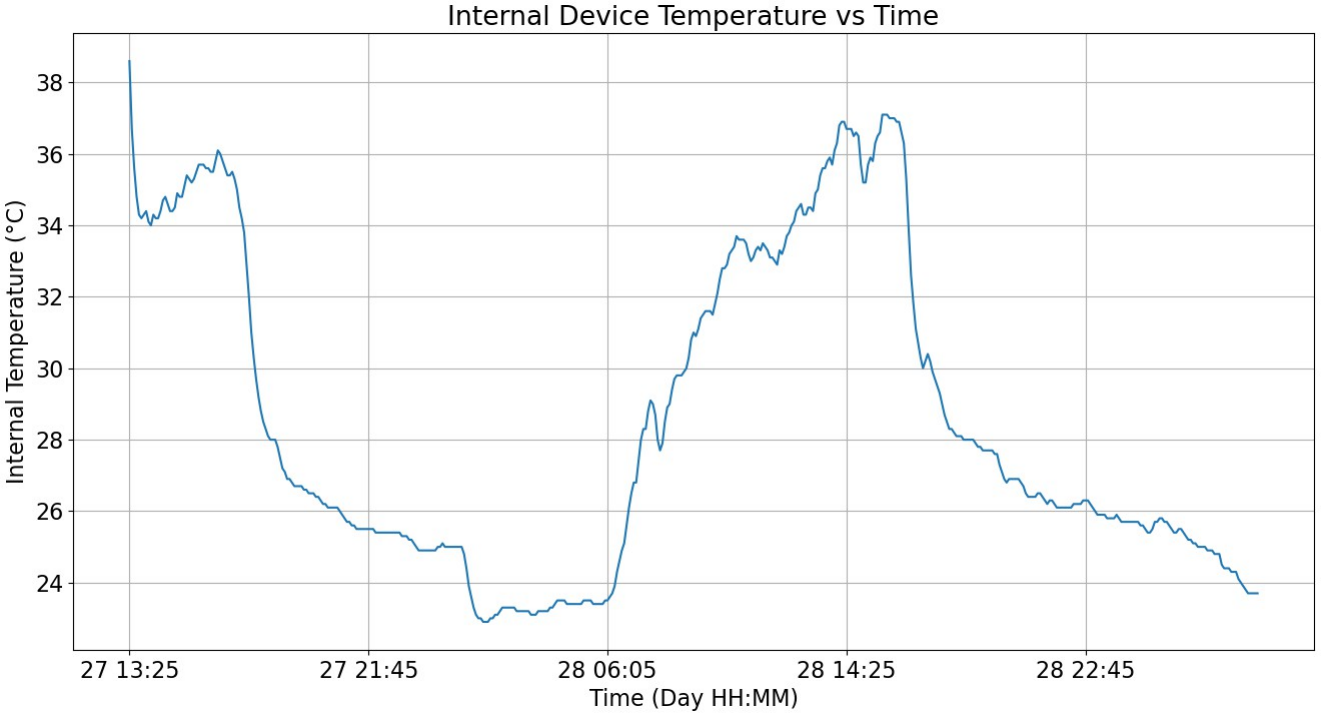
Internal device temperature data. Graph shows the first 40 hours of the field test.

An RC-5+ USB temperature data logger was placed inside the electronics box during the week-long test to record the temperature inside. This device measures temperatures within the range from -30°C to 70°C with an accuracy of ± 0.5°C between -20°C and 40°C and ±1.0°C otherwise. This was done to study the temperature behavior inside the electronics box. Fig 11 shows the recorded data.

#### Data collection

**Fig 12:**
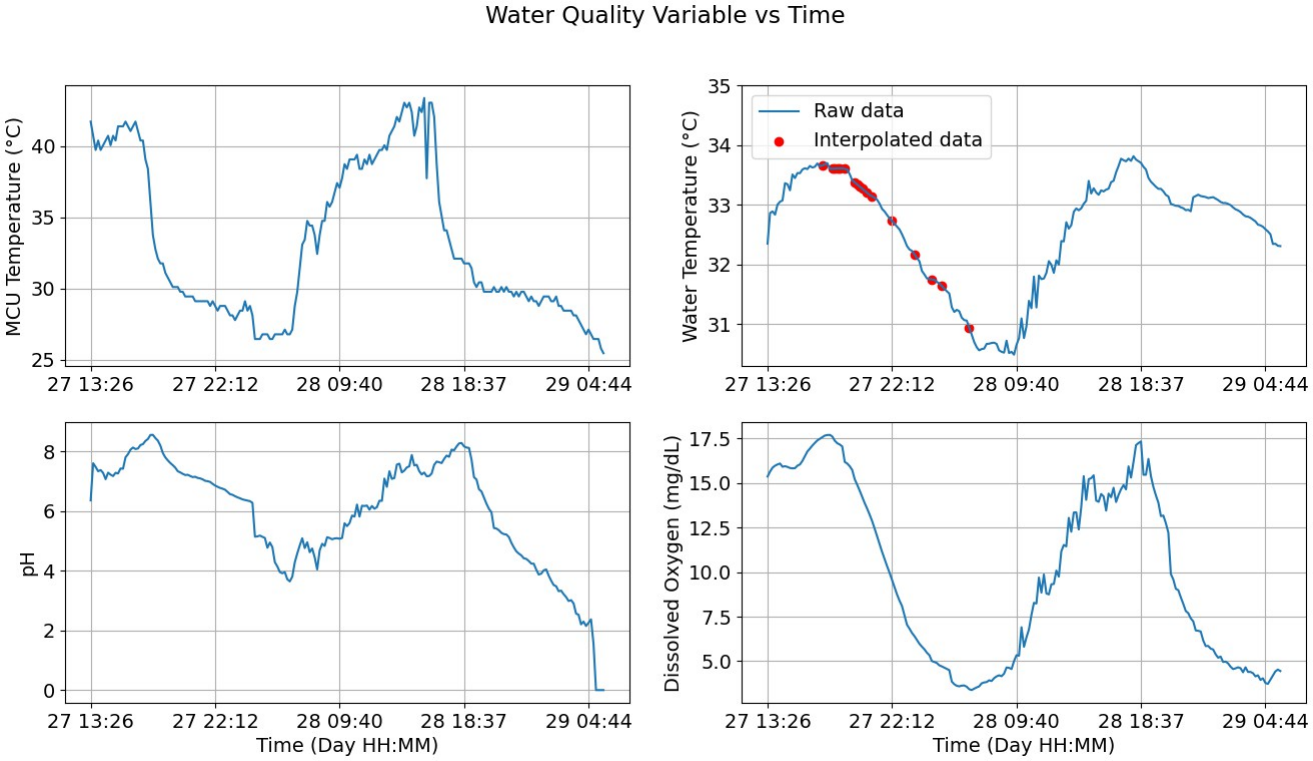
Data recollected with the device during the first 40 hours of the field test. The blue lines plot the raw data collected from the sensors. The red dots for the temperature values indicate replaced error values through a linear interpolation.

Both pH and water temperature can be sampled daily or after specific events such as checking the liming response. However, critical dissolved oxygen values can occur during the night and sampling at an hourly interval can help anticipate dangerous values. Thus, the maximum sampling interval was considered as one hour [1]. However, to account for possible packet loss during transmission and errors during the data reception from the sensor circuits, a faster sampling interval of 10 minutes was chosen for the field test. In addition, an internal microcontroller temperature reading was added to study the variation and verify that the operating range for the microcontroller was satisfied. Fig 12 plots the recorded data. Note that a few values received from the water temperature sensor were identified as errors through software. These data points were computed through with a linear interpolation using neighboring data points.

## Discussion

### Prototype performance observations

Overall, the construction presented no significant issues, and the mechanical structure performed as expected. The electronics box protected the circuit from the environment for the seven days of the test, particularly from heavy rain. No damage occurred to any of the electronics. However, the electronics box and the polystyrene box presented traces of possible water condensation inside. The buoy maintained its position, indicating that the anchor worked as expected. Algae growth occurred at every point submerged in the water: the anchor, the protection tube, the sensor probes, and part of the fridge. Further analysis is required to assess the impact of algae growth on the flotation performance and stability for long-term usage of the device. No water sipped into the electronics box due to the rain, and the buoy continued to be stable in the correct position.

Three different kinds of data were recorded during the field test. First, the coverage map points were taken to verify if the gateway location was capable of receiving data from the planned data buoy deployment site. This test was successful, and thus the data buoy was able to transmit data from the pond. Second, the internal temperature data recorded show that the maximum temperature achieved was around 38°C. This test helps verify if the internal device temperature stays inside the operating temperature given by the electronics. In this case, the batteries used limit the maximum temperature to 54°C [16]. Since the maximum temperature was around 38°C, this stays inside the operating limits. Finally, the three water quality variables corresponding to water temperature, pH and dissolved oxygen were recorded for 40 hours alongside the microcontroller’s internal temperature. Here, the daily variation of each variable can be observed, with lower values during the night that rise during the day [1]. For the microcontroller temperature, the values stay well inside the operating range of -40°C to 85°C [17]. Notably, each of the five variables, including the water quality and internal device temperature data, show a dip beginning on midnight of the day 28th. This event may be related to environmental variations, such as rain. The recorded data indicate that signal filtering for the water quality variables and error detection for device maintenance, recalibration and malfunction are required.

### Challenges and limitations

One of the first challenges encountered was the placement of the LoRaWAN gateway. The plan was to place the device inside a small building with Wi-Fi and an electrical plug. However, the building’s walls and roof were constructed from aluminum tiles. This type of structures could affect the transmission range and considerably reduce it. Future work could use an outdoor gateway placed in a better position.

The NUCLEO-L476RG board used in the node presented an additional limitation. Before entering Standby mode, the device must save the LoRaWAN session data in the Flash memory. After waking up, the device retrieves the data from the Flash memory and uses it to transmit the newly acquired measurements. The problem with this solution is that Flash memory has a low number of Read/Write cycles before it degrades. This number is minimum 10,000 cycles for the L4 board [17], which means that the prototype may damage the Flash memory and stop working during typical operation if left on for an extended amount of time. A better alternative would be to use a microcontroller with included EEPROM memory, such as an STM32L073RZ, which have an endurance of 100,000 cycles [18] and would last much longer before degrading. The STM32L476RG microcontroller inside the board does not include EEPROM memory. Therefore, a possible improvement to the prototype is to change the microcontroller to another with EEPROM memory and modify the program to save the session here instead of the Flash memory.

Table 2 shows a summary of the prices for each section of the data buoy prototype. Refer to the project website for more details in the corresponding Bill of Materials. The total price corresponds to close to 2.5 times the minimum wage in Colombia. Remarkably, close to 85% of the total cost is related to the sensors. This price includes the sensor probes, the reading circuits, and the required connectors and adapters. As a result, the biggest challenge for reducing the cost of these devices involves lowering those related to the sensors.

**Table 2:** Data buoy price summary in US dollars.

Section	Total Price (USD)	Percentage of Total Price (%)
Mechanical Structure	$ 24.30	3.69%
Electronics	$ 73.10	11.10%
Sensors	$ 561.38	85.21%
Complete Prototype	$ 658.79	100.00%

Also, more recent microcontrollers from the STM32WL series integrate a sub-GHz radio for LoRaWAN and Sigfox communications. These may prove to be a more appropriate alternative, and future work could investigate the potential of these newer models for remote monitoring.

## Conclusion

This paper presents an open-source prototype design for remote monitoring of water quality variables for fish farming. The device was tested at an operating fish farm, and future possible improvements based on issues encountered during the field test are proposed. The results successfully demonstrate the potential for battery-operated systems using LoRaWAN to replace conventional manual sampling methods.

## Supporting information

S1 Video Field test fish farm aerial view. (MOV)

S2 Video Data buoy prototype during operation. (MOV)
